# The effects of low dose X-irradiation on osteoblastic MC3T3-E1 cells in vitro

**DOI:** 10.1186/1471-2474-13-94

**Published:** 2012-06-08

**Authors:** Wei Xu, Lan Xu, Ming Chen, Yong Tao Mao, Zong Gang Xie, Shi Liang Wu, Qi Rong Dong

**Affiliations:** 1 Department of Orthopedics, The Second Affiliated Hospital of Soochow University, Suzhou, 215004, China; 2 Department of Biochemistry and Molecular Biology, School of Medicine, Soochow University, Suzhou, 215123, China

**Keywords:** Low dose irradiation, Osteoblasts, Proliferation, Differentiation

## Abstract

**Background:**

It has been indicated that moderate or high dose of X-irradiation could delay fracture union and cause osteoradionecrosis, in part, mediated by its effect on proliferation and differentiation of osteoblasts. However, whether low dose irradiation (LDI) has similar roles on osteoblasts is still unknown. In this study, we investigated whether and to what extent LDI could affect the proliferation, differentiation and mineralization of osteoblasts in vitro.

**Methods:**

The MC3T3-E1 cells were exposed to single dose of X-irradiation with 0, 0.1, 0.5, 1.0 Gy respectively. Cell proliferation, apoptosis, alkaline phosphatase (ALP) activity, and mineralization was evaluated by methylthiazoletetrazolium (MTT) and bromodeoxyuridine (BrdU) assay, flow cytometry, ALP viability kit and von Kossa staining, respectively. Osteocalcin (OCN) and core-binding factor α1 (Cbfα1) expressions were measured by real time-PCR and western blot, respectively.

**Results:**

The proliferation of the cells exposed to 2.0 Gy was significantly lower than those exposed to ≤1.0 Gy (p < 0.05) from Day 4 to Day 8, measured by MTT assay and BrdU incorporation. For cells exposed to ≤1.0 Gy, increasing dosages of X-irradiation had no significant effect on cell proliferation and apoptosis. Importantly, LDI of 0.5 and 1 Gy increased ALP activities and mineralized nodules of MC3T3-E1 cells. In addition, mRNA and protein expressions of OCN and Cbfα1 were also markedly increased after treatment with LDI at 0.5 and 1 Gy.

**Conclusions:**

LDI have different effects on proliferation and differentiation of osteoblasts from those of high dose of X-irradiation, which might suggest that LDI could lead to promotion of frature healing through enhancing the differentiation and mineralization of osteoblasts.

## Background

Ionizing radiation was a common therapy in the treatment of cancer, especially the head and neck carcinomas. Despite of its therapeutic value, osteoradionecrosis was considered to be one of the most serious clinical complications after radiation therapy [1]. However, as we know, the dosage of ionizing radiation was usually moderate or high in the application of cancer therapy.

Clinically, patients with fractures were often exposed to CT scan before surgery, fluoroscopy during operation and X-ray during follow-up postoperatively, where they received irradiation dose usually ≤1 Gy [2-4]. It meant that injured bone tissues might exist when exposed to low dose irradiation (LDI). But there were limited studies on the effects of LDI on the healing and remodeling of bone tissues. To understand this, our preliminary studies surprisingly showed that LDI could promote fracture mineralization in Sprague–Dawley rat model [5]. It was generally considered that ordered proliferation and differentiation of osteoblast was indispensable for mineralization of extracellular matrix in bone formation during wound healing [6]. To our knowledge, LDI had several biologic effects of increasing expression of vascular endothelial growth factor (VEGF) and mobilization of progenitor cells [7,8].

In the light of this, the major objective of this study was to explore the molecular mechanism of effects of LDI on healing and remodeling of bone tissues, and to examine whether and to what extent LDI could influence the proliferation and differentiation processes of the osteoblastic-like cell line (MC3T3-E1). As a consequence, our findings would be beneficial for further understanding the underlying cellular and molecular mechanisms of the potential roles of LDI on fracture healing.

## Methods

### Cell culture

MC3T3-E1 cells were provided by the Institute of Biochemistry and Cell Biology, China. The cells were cultured in α-MEM medium consisting of 10% FBS, 5 mM β-glycerophosphate, 50 μg/ml L-ascorbic acid (Sigma, USA) as described by Yamasaki et al [9]. The culture medium was changed every three days. After cells had reached 70% confluence, cells were detached by treatment with 0.05% trypsin, and replated for experiments. Low passage frozen stocks were prepared and early passage cells were used in the experiments (less than passage 10).

### Irradiation of osteoblastic cells

MC3T3-E1 cells were irradiated respectively with 0 (as the control), 0.1, 0.5 and 1.0 Gy X-irradiation (at a dose rate of 200 cGy/min) by a medical linear accelerator with a 6 MV radiation source (Siemens Primus, Concord, CA, USA) on the next day after being seeded (day 0).

### Proliferation assay

Cells were plated at a density of 1 × 10^3^ cells/well into 96-well plates for cell growth assay. The methylthiazoletetrazolium (MTT, Sigma) assay was performed from Day 2 to Day 8 as described by Carmichael et al. [10]. In brief, 20 μl MTT (5 mg/ml) was added to the wells and the plate was incubated at 37°C for 4 h. Subsequently, 100 μl of dimethyl sulphoxide was added to release the formed formazan crystals from the living cells’ mitochondria into the solution. Optical density (OD) was measured at 495 nm and automatically calculated as absorbance using the microplate scanning spectrophotometer (POWERWAVE.XS, Bio-Tek, USA).

Cell proliferation was also determined by bromodeoxyuridine (BrdU) incorporation analysis. Cells were plated in 96-well plates (1 × 10^3^ cells/well) and the BrdU incorporation in the new synthesized DNA was quantified as described by Kanazawa et al. [11]. Briefly, BrdU (Roche, Germany) was added to the medium for 2 h. After removal of the culture medium, the cells were fixed and the DNA was denatured. Anti-BrdU antibody was then added to measure the amount of incorporated BrdU. Absorbance of each well was measured using the microplate scanning spectrophotometer at 450 nm.

### Apoptosis analysis

Cells were plated at a density of 5 × 10^4^ cells/well into 6-well plates. Apoptosis of MC3T3-E1 cells were evaluated by flow cytometery. Cells were collected and detected using the annexin V-FITC/PI apoptosis detection kit (Molecular Probes, USA) as described by Xu et al. [12]. In brief, cells were collected and resuspended in 1 × cold binding buffer (10 mM Hepes, pH 7.4, 150 mM NaCl, 2.5 mM CaCl_2_, 1 mM MgCl_2_, 4% BSA) for analysis. Cells were also stained with PI to detect late apoptosis cells. 10,000 cells were subjected to flow cytometric analysis. 

### Assay of alkaline phosphatase activity and alkaline phosphatase staining

Cells were plated at a density of 2 × 10^4^ cells/well into 24- well plates. The cells were cultured for 7 days after irradiation, rinsed three times with PBS, and 600 μl of distilled water were added to each well and sonicated. The protein assay was performed with the bicinchoninic acid (BCA) protein assay reagent (Sigma). ALP activity was assayed by a method modified from that of Lowry et al. [13]. In brief, the assay mixtures contained 0.1 M 2-amino-2-methyl-1-propanol, 1 mM MgCl_2_, 8 mM p-nitrophenyl phosphate disodium, and cell homogenates. After incubation for 4 min at 37°C, the reaction was stopped with 0.1 N NaOH, and the absorbance was read at 405 nm. Each value was expressed as p-nitrophenol produced in nanomoles per minute per microgram of protein. 

Similarly, ALP staining was also performed by a standard protocol on day 7 after irradiation. In brief, cells were fixed in 100% methanol and overlaid with 1.5 ml of 0.15 mg/ml 5-bromo-4-chloro-3-indolyphosphate plus 0.3 mg/ml nitroblue tetrazolium chloride in 0.1 M Tris–HCl, pH 9.5, 0.01 N NaOH, and 0.05 M MgCl_2_, followed by incubation at room temperature for 2 h in the dark.

### Assay of mineralization

The mineralization of MC3T3-E1 cells was determined in 6-well (1 × 10^5^ cells/well) using von Kossa staining on day 14 after irradiation. Cells were added by 1% silver nitrate and put in the dark place for 45 min. Then, cells were washed for 10 min and added by 95% sodium thiosulfate to react for 5 min, fixed with 95% ethanol for 10 min. Mineralized nodules stained with dark brown to black dots were counted at 30 × magnification using a dissecting microscope by placing the culture plate upon a transparent acetate grid ruled in 2 mm squares as described by Bellows et al [14].

### Real-time PCR quantification for mRNA expression of osteocalcin (OCN), core-binding factor α1 (Cbfα1)

Cells were seeded at a density of 1 × 10^5^ cells/well into 6-well plates. On day 10 after irradiation, the total RNA was extracted with Trizol reagent (Gibco, USA) and cDNA was synthesized with the reverse transcription kit (TakaRa, China). SYBR green chemistry (Toyobo, Japan) was used to perform quantitative determination for the mRNAs for OCN, Cbfα1, β-actin was used as endogenous control. Primer sequences were showed as followed: OCN forward primer, 5′-CTGGCTGCGCTCTGTCTCT-3′; reverse primer, 5′- TGCTTGGACATGAAGGCTTTG -3′. Cbfα1 forward primer, 5′- AAGTGCGGTGCAAACTTTCT -3′; reverse primer, 5′- TCTCGGTGGCTGGTAGTGA -3′. β-actin forward primer, 5′- CTGGCACCACACCTTCTACA -3′; reverse primer, 5′- GGTACGACCAGAGGCATACA -3′. Analysis was performed with ABI PRISM 7000 (PE Applied Biosystems Inc). Reaction condition was 95°C for 15 min, 40 cycles of denaturation at 94°C for 15 s, and annealing and extension at 60°C for 1 min. The mRNA expression of OCN and Cbfα1 was normalized to endogenous control and relative to a calibrator, and was calculated using formula as described by Livak et al. [15]. Results were expressed as fold change in gene expression relative to the control group (0 Gy).

### Western-blot analysis for protein expression of OCN, Cbfα1

Cells were seeded at a density of 1 × 10^5^ cells/well into 6-well plates and the irradiated cells were cultured steadily for 10 days. Cells were incubated with 300 μl of lysis buffer (1% NP-40, 0.5% deoxycholate, 0.1% SDS) on ice for 30 min. The lysate was centrifuged at 14000 rpm, the supernatant was collected and protein concentration was determined by the Bicinchoninine acid assay, meanwhile the standard curve was mapped. Equally, 20 μg of crude protein extracts for every sample were loaded to 10% sodium dodecyl sulphate–polyacrylamide gels, and then transferred onto nitrocellulose membranes. The membrane was incubated overnight with primary monoclonal antibodies rabbit anti-mice OCN (diluted 1:1000, R&D, USA), Cbfα1 (diluted 1:1000, R&D, USA) and β-actin (diluted 1:800, Santa Cruz, USA). After washing three times, the samples were continually incubated with the HRP-conjugated goat anti-rabbit IgG-AP (1:2000) for 60 min. The protein was visualized using the BM Chemiluminescence Western Blotting Kit (Boehringer, Mannheim, Germany) according to the manufacturer’s protocol.

### Statistical analysis

Each experiment was repeated independently three times and all data were expressed as mean ± SD. Analyses of variance (one-way ANOVA) were performed using SPSS 17.0. P value less than 0.05 was considered significant.

## Results

### Effects of irradiation on proliferation of MC3T3-E1 cells

**Figure 1:**
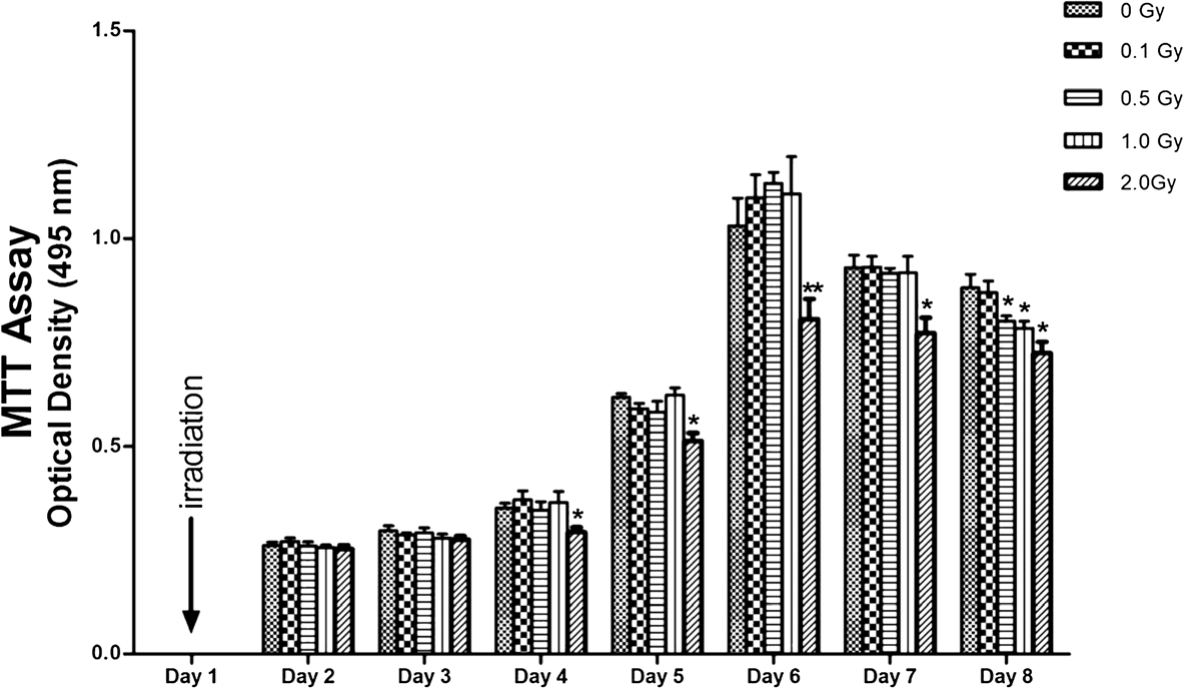
**Growth curve of irradiated MC3T3-E1 cells assessed by MTT assay.** MC3T3-E1 cells were exposed to 0, 0.1, 0.5, 1.0 and 2.0 Gy of X-ray irradiation respectively. The effect of irradiation on cell growth was assessed by MTT assay at 24-h intervals until day 8 after been seeded. Optical density (OD) was measured at 495 nm and data were expressed as the mean ± SD of triplicate experiments (n = 3). From day 4 to day 8, relative proliferation rate of the cells exposed to 2.0 Gy was significantly lower than those of the control group (0 Gy) (* p < 0.05, ** p < 0.01). On day 8, relative proliferation rates of the cells exposed to 0.5 and 1 Gy were lower than those of the control group and 0.1 Gy group(* p < 0.05).

**Figure 2:**
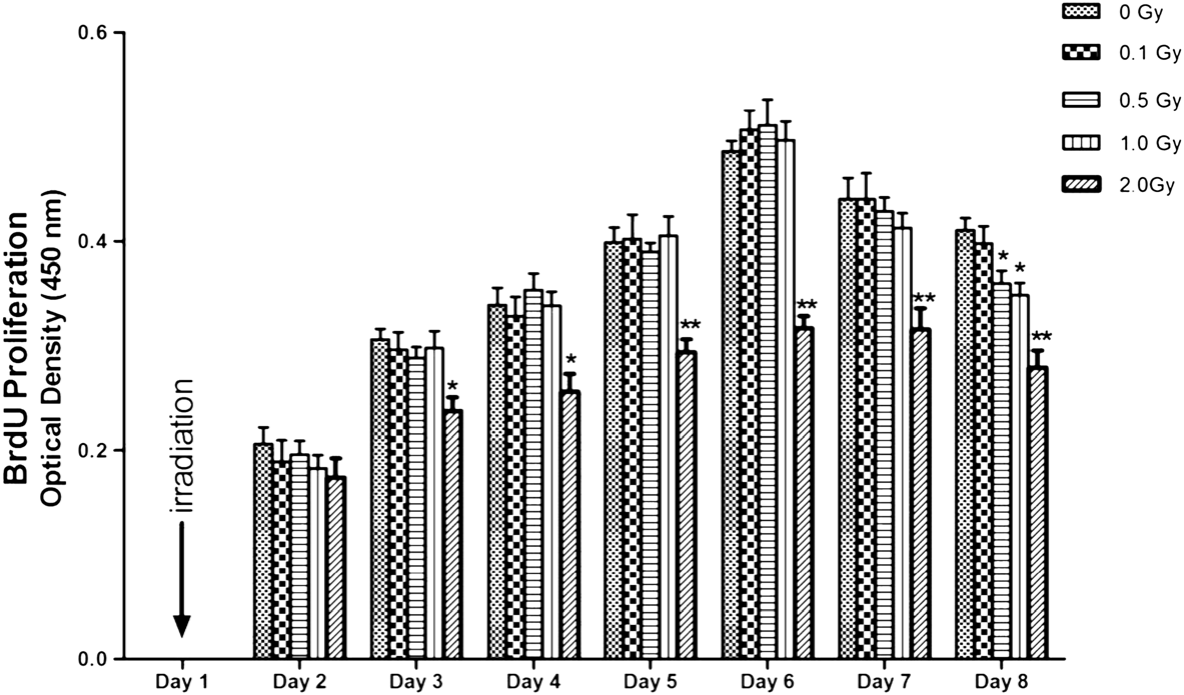
**Proliferation of MC3T3-E1 cells after irradiation assessed by BrdU incorporation assay.** MC3T3-E1 cells were exposed to 0, 0.1, 0.5, 1.0 and 2.0 Gy of X-ray irradiation respectively. Cell proliferation was determined by BrdU incorporation assay at 24-h intervals until day 8 after been seeded. Absorbance in each well was measured at 450 nm and data were expressed as the mean ± SD of triplicate experiments (n = 3). From day 3 to day 8, the DNA synthesis of the cells exposed to 2.0 Gy was significantly lower than those of the control group (0 Gy) (* p < 0.05, ** p < 0.01). On day 8, the DNA synthesis of the cells exposed to 0.5 and 1 Gy were lower than those of the control group and 0.1 Gy group(* p < 0.05).

The relative proliferation rate of the cells exposed to 2.0 Gy was significantly lower than those exposed to ≤1.0 Gy (p < 0.05) from Day 4 to Day 8, measured by MTT assay. For cells exposed to ≤1.0 Gy, Increasing dosages of X-irradiation had no effects on relative proliferation rates of MC3T3-E1 cells from day 2 to day 7. On day 8, relative proliferation rate of cells exposed to 0.5 and 1 Gy was lower than that of the control and 0.1 Gy groups (p < 0.05) (Figure 1). Interestingly, cell proliferation was reduced on Day 8 when compared to Day 6 (data not shown). The BrdU uptake results was similar to MTT assay (Figure 2).

### Effects of irradiation on apoptosis of MC3T3-E1 cells

**Figure 3:**
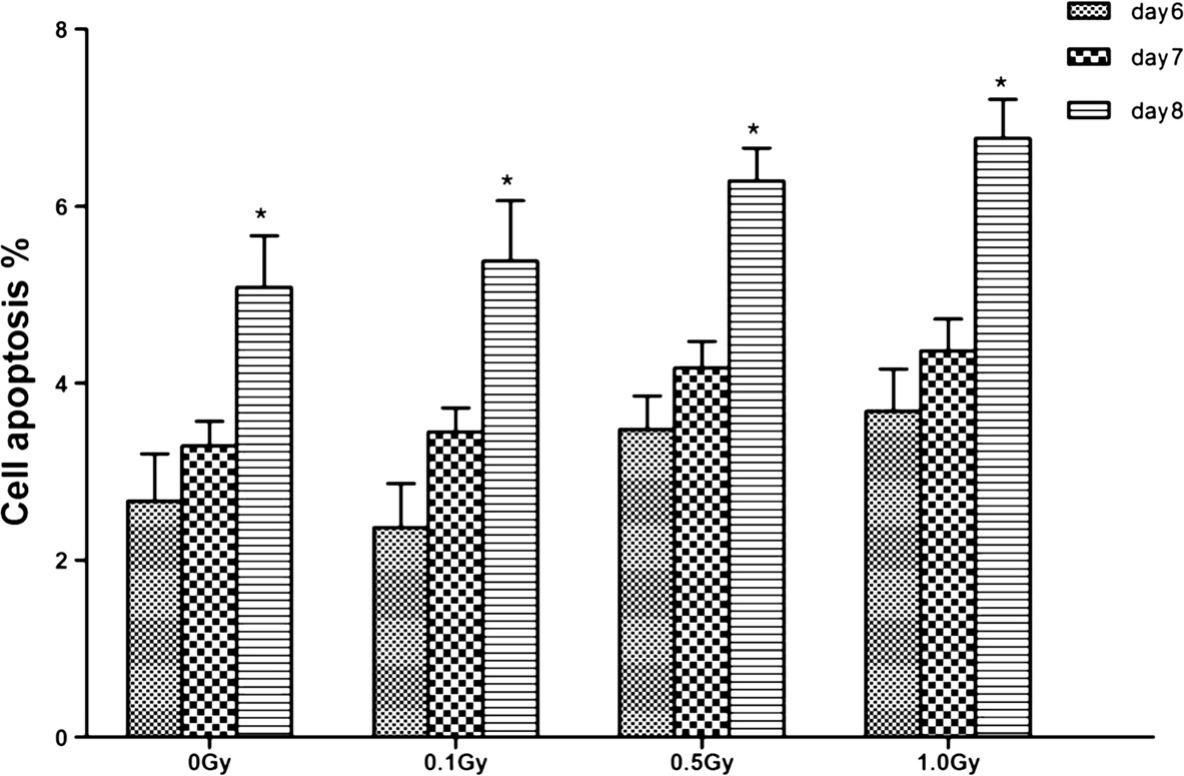
**Apoptosis of irradiated MC3T3-E1 cells on day 6–8 after been seeded.** MC3T3-E1 cells were exposed to 0, 0.1, 0.5, 1.0 Gy of X-irradiation. Cells on day 6–8 were double stained with annexin-V-FITC and PI and analyzed by flow cytometry. Data were expressed as the mean ± SD of triplicate experiments (n = 3). For each dose, apoptosis cells (including early apoptotic and late apoptotic cells) was gradually increased from day 6 to day 8 and the number of apoptosis cell on day 8 was significantly more than that on day 6 (p < 0.05).

To ascertain whether proliferation changes on day 6–8 were linked to apoptosis, we evaluated cell apoptosis. Data of apoptosis showed that there was no statistically significant difference among groups exposed to ≤1.0 Gy irradiation (data not shown). But for each dose, number of apoptotic cells (including early apoptotic and late apoptotic cells) on day 8 was significantly more than that on day 6 (p < 0.05) (Figure 3).

### Effects of irradiation on ALP activity and mineralization of MC3T3-E1 cells

**Figure 4:**
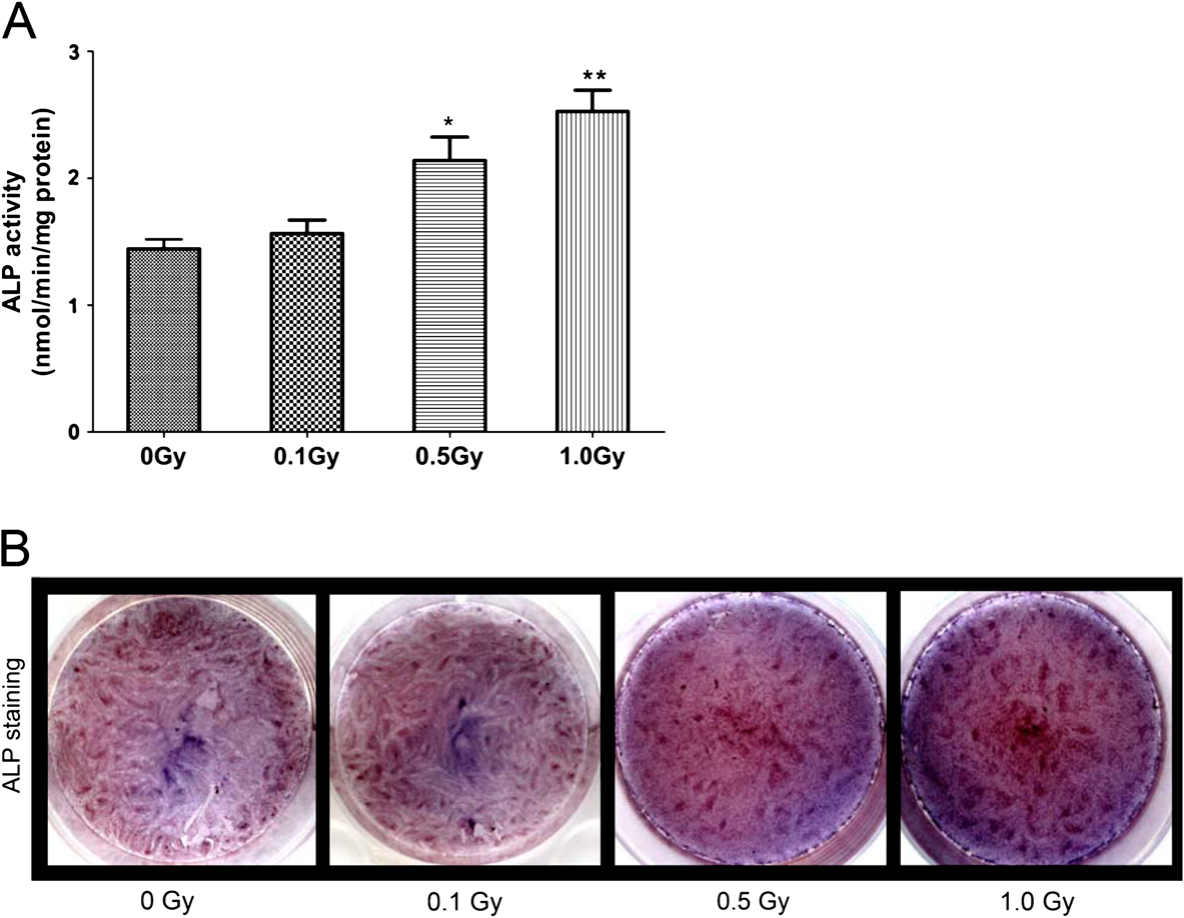
**Effects of X-irradiation on ALP activity and staining of MC3T3-E1 cells.** MC3T3-E1 cells were exposed to 0, 0.1, 0.5 and 1.0 Gy of X-irradiation respectively and cells were cultured for 7 days after irradiation. (**A**) ALP activity was measured and the data were expressed as mean ± SD (n = 3). The ALP activity of 0.5 Gy and 1.0 Gy groups was significantly increased than that of the control group (0 Gy) (*p < 0.05, **p < 0.01). However, 0.1 Gy groups had no significant difference compared with the control. (**B**) represented plate view of ALP staining. There were more positive cells in 0.5 and 1.0 Gy groups compared with the control.

**Figure 5:**
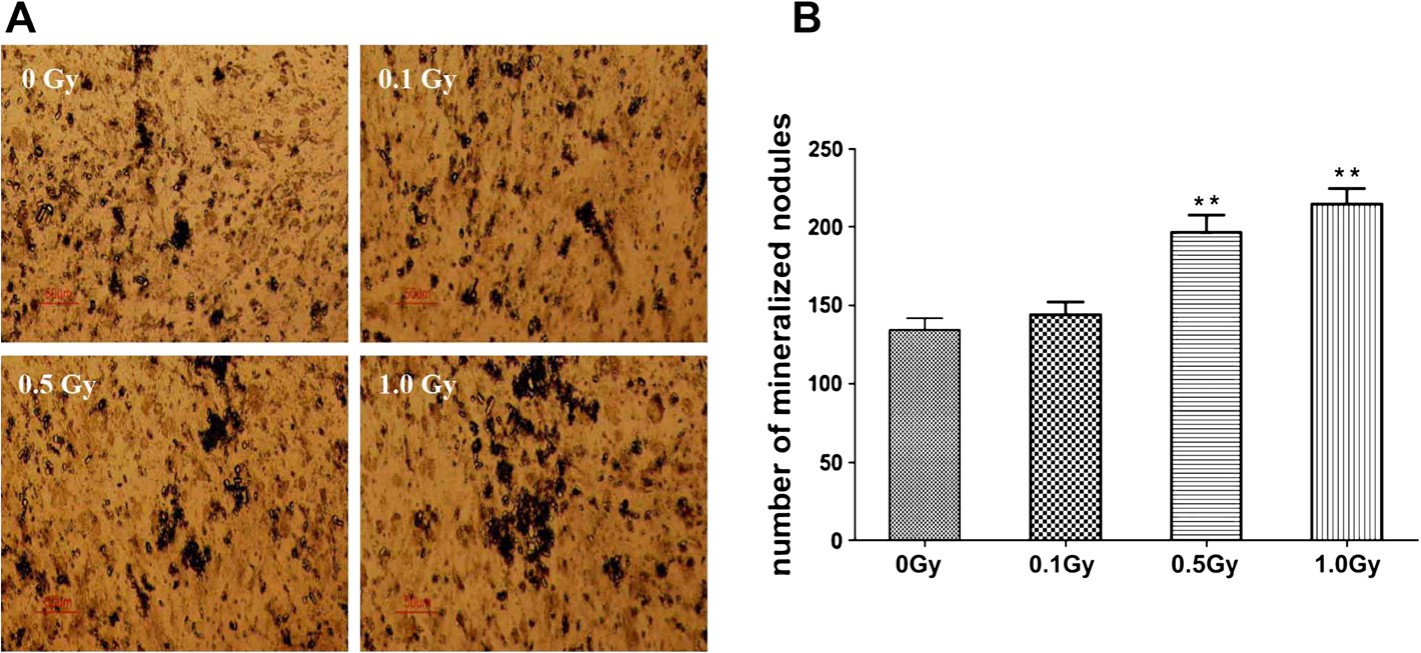
**Effects of X-irradiation on mineralized nodules of MC3T3-E1 cells.** MC3T3-E1 cells were exposed to 0, 0.1, 0.5 and 1.0 Gy of X-irradiation respectively and von Kossa staining was performed on day 14 after irradiation. **A** represented microscope images. The number of mineralized nodules of osteoblasts was counted and shown in **B**. Data were expressed as mean ± SD of 3 wells. Significant difference in 0.5 and 1.0 Gy groups was observed compared with the control group (0 Gy) (**p<0.01).

ALP activity and von Kossa staining were performed on day 7 and day 14 after irradiation, respectively. The ALP activity of 0.5 Gy and 1.0 Gy groups was significantly increased than that of the control and 0.1 Gy groups (p < 0.05). However, 0.1 Gy group had no significant difference compared with the control (Figure 4). Similarly, ALP staining showed more positive cells (cytosol red coloration) in 0.5 Gy and 1.0 Gy groups compared with the control and 0.1 Gy groups (Figure 4B). von Kossa staining showed that the number of mineralized nodules in 0.5 Gy and 1.0 Gy group remarkably increased compared with the control group (p < 0.05). The number of mineralized nodules had no significant difference between 0.1 Gy group and the control group (Figure 5A, B).

### Effects of irradiation on expression of OCN and Cbfα1

**Figure 6:**
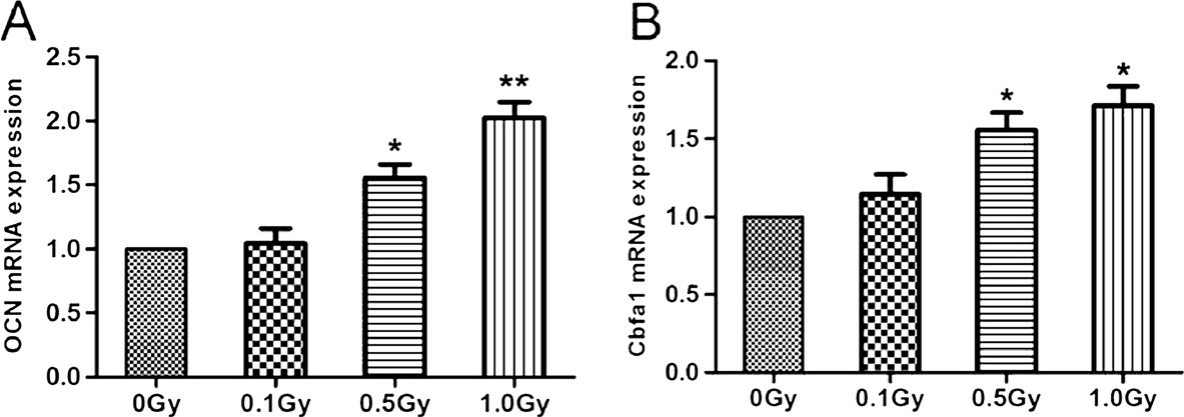
** OCN, Cbfα1 mRNA expression of irradiated MC3T3-E1 cells.** MC3T3-E1 cells were exposed to 0, 0.1, 0.5 and 1.0 Gy of X-irradiation respectively and cells were harvested on day 10 after irradiation. Total RNA was collected and real-time PCR was performed, β-actin as an endogenous control. Results were expressed as fold change over the control group (0 Gy). (**A**) OCN mRNA expression in 0.5 and 1 Gy groups were significantly increased compared with the control group (*p < 0.05, **p < 0.01). (**B**) Cbfα1 mRNA expression in 0.5 and 1 Gy groups were significantly increased compared with the control group (*p < 0.05).

**Figure 7:**
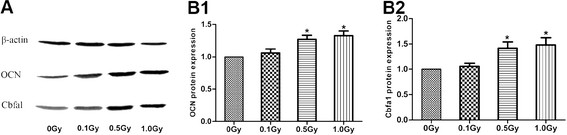
**OCN, Cbfα1 protein expression of irradiated MC3T3-E1 cells.** MC3T3-E1 cells were exposed to 0, 0.1, 0.5 and 1.0 Gy of X-irradiation respectively and cells were harvested on day 10 after irradiation. (**A**) The protein productions of OCN and Cbfα1 were detected by Western blot with β-actin as an endogenous control. (**B**) The quantitative date of OCN (**B1**) and Cbfα1 (**B2**) was band-density ratios compared to the control group. X-irradiation of 0.5 and 1 Gy resulted in obviously increase of protein level of OCN and Cbfα1 (*P < 0.05).

To ascertain the effects of LDI on differentiation of MC3T3-E1 cells, we examined gene expression associated with osteoblast differentiation on day 10 after irradiation by real-time PCR. Real-time PCR showed mRNA expression of OCN and Cbfa1 was up-regulated after irradiation at 0.5 Gy and 1.0 Gy (p < 0.05) (Figure 6A, B). To verify the results of mRNA expression of OCN, Cbfα1, we further analyzed the protein expression of OCN and Cbfα1. OCN and Cbfα1 protein expression were generally consistent with their mRNA expression, and X-irradiation of 0.5 and 1 Gy resulted in obvious increase of protein level of OCN and Cbfα1 (p < 0.05) (Figure 7A, B).

## Discussion

Various clinical and experimental investigations showed that irradiation could affect osteoblastic activity, including proliferation decrease, cell cycle arrest, increased sensitivity to apoptosis, and reduce of osteoblast differentiation, which was tightly associated with fracture union delay and osteoradionecrosis. However, radiation dose selected above was mainly moderate or high [16-24]. This study focused on effects of low dose X-irradiation on proliferation, differentiation and mineralization of osteoblasts (MC3T3-E1 cells) in vitro.

Above all, we tried to determine the nontoxic doses of radiation on MC3T3-E1 osteoblastic cells. We found that cell proliferation measured by MTT and BrdU was significantly decreased when cells were exposed to 2.0 Gy, which is consistent with some other studies [25,26], ruling out the possible severe toxicity of radiation used in the present study at low dose X-irradiation (≤ 1 Gy).

Cell proliferation dropped significantly on Day 8. Coincidently, we found that cell apoptosis increased instead at this time. Furthermore, we considered that cell reached confluence and contact inhibition of cell-to-cell had initiated at this stage. On the other hand, cell proliferation decreased in 0.5 Gy and 1.0 Gy groups on Day 8. MC3T3-E1 cells showed alkaline phosphatase activity here and began to differentiation. Some studies reported that proliferation activity was inversely related to differentiation of osteoblasts [27,28]. Thus, we supposed that proliferation decrease might be associated with their promoting cell differentiation.

It was widely accepted that ALP was the early phenotypic marker and in accordance with the differentiation of osteoblasts [29,30]. Formation of mineralized nodules was the ultimate expression of the osteogenic phenotype in vitro and the characteristic marker of mineralization [31]. Our results showed that ALP activity and mineralized nodules exposed to 0.1 Gy were not statistically different from those of nonirradiated group, which may represent a threshold effect for X-ray irradiation. But in LDI of 0.5 Gy and 1.0 Gy groups, ALP activity and mineralized nodules were positively correlated with the dosage of irradiation.

To further ascertain the effects of LDI on differentiation of MC3T3-E1 cells, we examined the expression of genes and proteins associated with osteoblastic differentiation. OCN was generally considered as a late marker in the mineralization stage, which bond with calcium and hydroxyapatite closely [32,33]. Our results showed that OCN expression was increased in 0.5 and 1.0 Gy groups compared with that of control. We also analyzed gene expression of collagen I. Surprisingly, no change was found in collagen I (data not shown). Wang reported that MC3T3 subclones with both high and low differentiation potential produced similar amounts of collagen in culture [34]. Variety of researches demonstrated that osteoblasts expressed the nuclear protein Cbfα1, which could act as a transcriptional factor and bind with certain cis-acting elements of OCN genes to further enhance their transcriptional activities [35,36]. The skeletal systems of the mice with a homozygous mutation in Cbfα1 showed a complete lack of ossification [37]. Cbfα1-deficient calvarial cells did not acquire osteoblastic phenotypes [38]. Thus, Cbfα1 was a critical gene not only for osteoblast differentiation but also for osteoblast function. Consequently, X-irradiation of 0.5 Gy and 1.0 Gy could increase the expression of Cbfα1, which might further activate the transcriptional activities of OCN in MC3T3-E1 cells.

Taking all results into consideration, low dose X-irradiation promoted differentiation of osteoblasts, but without impairing proliferation. As multipotential cells, mesenchymal stem cells (MSCs) could be induced into osteoblasts and had been long taken as important subjects of research. Among studies of moderate and high dose irradiation, some showed that radiation mainly suppressed the proliferation or cell cycle progression [39], while some showed that only the process of differentiation was suppressed [40,41], as well as some showed that proliferation and differentiation of MSCs were both suppressed [42,43]. Multipotential cells are heterogeneous in differentiation potential and comprise both progenitors and relative mature cells. These controversial conclusions may be associated with MSCs themselves besides the diversity of radiation dose and research models.

Few available literature described the effects of low-dose irradiation on osteoblasts in vitro. Dare [44] reported that ≤400 mGy of X-irradiation had no significant changes in proliferation and differentiation of MC3T3-E1 cells. Ahmad [26] showed ≤2 Gy of 137Cs irradiator had no effects on proliferation and ALP activity of human fetal osteoblast 1.19 cells. Kurpinski [45] reported that 1 Gy X-ray perturbed DNA replication and DNA binding activity of MSCs, without impairing their osteogenic differentiation process in vitro. The discrepancy needed further study and the difference in radio sources, cell types and timing was also undoubtedly important like Kurpinski and Jin reported [45,46].

Interestingly, irradiation also induced terminal differentiation in some other culture systems, such as, human skin fibroblasts [47] and erythroid progenitor cells [48]. While some reported ionizing radiation greater than 2 Gy promoted osteoblasts terminal differentiation [18,23]. Different from the effects of moderate and high dose irradiation, LDI had no impacts on the process of proliferation of osteoblasts in our study. We thought that increasing differentiation of osteoblasts by LDI might be regarded as a kind of promotion of tissue repair on the condition that osteoblasts have the normal ability of proliferation. Furthermore, the more differentiated osteoblasts can help callus formation and callus calcification in vivo.

## Conclusion

This study indicated that LDI could enhance the differentiation and mineralization of MC3T3-E1 cells, without affecting proliferation at the early stage. Thus we hypothesized that LDI would be beneficial for healing of injured bone tissues.

Fracture healing is a complex biologic phenomenon. We are aware of the limitations of an in vitro study and don’t draw general conclusion only from a cell line (MC3T3-E1) cells. In addition, we examined the response of osteoblasts to single low dose irradiation in the current experimental study. Future studies may also be needed to consider clinical situation where radiation is delivered in a fractionated manner. Fractionated irradiation and mechanical study should be further designed for better understanding the effects of LDI on osteoblasts.

## Abbreviations

LDI, low dose irradiation; MTT, methylthiazoletetrazolium; BrdU, bromodeoxyuridine; ALP, alkaline phosphatase; OCN, osteocalin; Cbfα1, core-binding factor α1.

## Competing interests

The study was supported by the nature science fund of Jiangsu Province in China (No. SBK200920031). The authors receive nothing of value and have no conflict of interest.

## Authors’ contributions

All authors contributed to this study. WX made contribution in conception and design of the study, participated in carrying out the experiments. LX carried out the experiments, collected the data and made the statistical analysis. MC carried out the experiments and helped to draft the manuscript. YTM and ZGX participate in the design and coordination of the study. SLW gave intellectual support and revised critically the manuscript. QRD led the project, gave intellectual support and drafted the manuscript. All authors read and approved the final manuscript.

## Pre-publication history

The pre-publication history for this paper can be accessed here:

http://www.biomedcentral.com/1471-2474/13/94/prepub
